# Marked differences in frequencies of statin therapy relevant SLCO1B1 variants and haplotypes between Roma and Hungarian populations

**DOI:** 10.1186/s12863-015-0262-4

**Published:** 2015-09-03

**Authors:** Agnes Nagy, Csilla Sipeky, Renata Szalai, Bela Imre Melegh, Petra Matyas, Alma Ganczer, Kalman Toth, Bela Melegh

**Affiliations:** 1st Department of Internal Medicine, University of Pecs, Pecs, Hungary; Department of Medical Genetics, Clinical Centre, University of Pecs, Szigeti 12, H-7624 Pecs, Hungary; Janos Szentagothai Research Centre, Human Genetic and Pharmacogenomic Research Group, University of Pecs, Pecs, Hungary

**Keywords:** SLCO1B1, Statin, Haplotype, Roma, Hungarian, Pharmacogenetics

## Abstract

**Background:**

SLCO1B1 polymorphisms are relevant in statin pharmacokinetics. Aim of this study was to investigate the genetic variability and haplotype profile of SLCO1B1 polymorphisms in Roma and Hungarian populations. Genotypes of 470 Roma and 442 Hungarian subjects for c.388A > G, c.521T > C and c.1498-1331T > C polymorphisms were determined by PCR-RFLP assay. Using these SNPs eight different haplotypes could be differentiated.

**Results:**

Differences were found between Roma and Hungarians in SLCO1B1 388AA (24.5 vs. 45.5 %), GG (33.4 vs. 17.9 %) genotypes, AG + GG (75.5 vs. 54.5 %) carriers, in G allele frequency (0.545 vs. 0.362), respectively (*p* < 0.001). The most common SLCO1B1 haplotype was the ht8 (GTT) both in Roma (43.6 %) and in Hungarian (59.1 %) samples. The ht6 (GCT) was not present in Roma population samples Haplotype analyses showed striking differences between the Roma and Hungarian samples in ht4 (ATT, 37.2 % vs 20.8 %), ht5 (GCC, 1.15 % vs. 3.62 %) and ht8 (GTT, 43.6 % vs. 59.1 %) haplotypes (*p* < 0.01), respectively. Linkage disequilibrium analysis showed that the studied variants are in different linkage disequilibrium patterns depending on the ethnic origin.

**Conclusions:**

Similarly to Caucasians the 388G is the minor allele in Hungarians, however, in Roma the 388A was found to be the minor allele contrary to Indians (India). The minor allele frequency of 521T > C and 1498-1331T > C SNPs are almost three times higher in Romas than in Indians (Singapore and Gujarati, respectively). Observed allele frequency for 1498-1331T > C polymorphism reflects the measured average European rates in Hungarians. The results can be applied in population specific treatment algorithms when developing effective programs for statin therapy.

## Background

Solute carrier organic anion transporter family member 1B1 (*SLCO1B1*) (alternative names OATP2, OATPC, LST1, SLC21A6) gene encodes for a membrane-bound sodium-independent organic anion transporter protein (OATP1B1) that is involved in active cellular influx of endogenous substrates (e.g. bile acids), xenobiotics and a wide panel of pharmaceutical compounds (e.g. statins, antibiotics, ACE inhibitors). OATP1B1 protein mediates active intracellular hepatic transport of anionic compounds, important in drug hepatic clearance and in general drug disposition [1]. General role of the uptake transporter is removal of substrates from the blood into the liver. Sequence variations play an important role in pharmaceutical response to a number of drugs [2].

*SLCO1B1* gene is located on chromosome 12 (15 exons, 190 common variants) and encodes a 691 amino acid protein with 12 transmembrane helices [3, 4]. OATP1B1 is expressed predominantly on the basolateral membrane of hepatocytes, where it mediates active intracellular hepatic transport of various anionic compounds [5, 6].

OATP1B1-dependent transport is an important step in mediating drug hepatic clearance. Statins (HMG-CoA reductase inhibitors) are widely prescribed for cardiovascular disease (CVD) risk reduction [1, 7]. OATP1B1 transport is particularly important for hepatic accessibility of pravastatin, as this compound is too hydrophilic to gain significant hepatocellular entry through passive transport [8]. OATP1B1-dependent transport could well be important for the acid (active) form of simvastatin, (and other statins less hydrophobic than pravastatin) as *SLCO1B1* variants were recently associated with simvastatin-induced myopathies [9], implying that OATP1B1 was involved with simvastatin transport. The *SLCO1B1* gene spans fifteen exons and 190 common variants with minor allele frequency greater than 5 % (http://hapmap.ncbi.nlm.nih.gov/). Of these, two common non-synonymous *SLCO1B1* variants have been well characterized: rs2306283 (388A > G, N130D) and rs4149056 (T521C, V174A) [10]. Recent reviews describe the role of OATP1B1 in general drug disposition [4] and, specifically, in statin pharmacokinetics [7]. Another recent discovery found the C variant of the rs4363657 polymorphism of SLCO1B1 gene strongly associated with statin induced myopathy in more than 60 % of cases [11]. The association with myopathy was underlined by several other studies with simvastatin and atorvastatin [9, 12]. However, there appears to be no increased risk of myalgia among users of rosuvastatin who carry the rs4363657C allele in SLCO1B1 [13].

Several studies indicate the wide substrate selectivity of OATP1B1 and demonstrate that sequence variation at the *SLCO1B1* locus may have a sizable impact on pharmaceutical. Consequently, importance of *SLCO1B1* genetic variants in clinical pharmacogenetics is underlined by the fact that there are some drugs (e.g. simvastatin) on the basis of the US Food and Drug Administration recommendations have already incorporated information regarding pharmacogenetic information about the *SLCO1B1* in their drug label (www.fda.gov). Extensive literature and FDA warning labels indicate increased risk for myopathy in patients with specific genetic differences on the SLCO1B1 gene. Guidelines regarding the use of pharmacogenomic tests in dosing for simvastatin have been published in Clinical Pharmacology and Therapeutics by the Clinical Pharmacogenetics Implementation Consortium (CPIC) [14].

Regarding the high clinical importance of *SLCO1B1* pharmacogene in drug dosing of statins, it is noteworthy to evaluate the *SLCO1B1* genotype not only of single patients, but also the general frequency of the polymorphisms in the whole populations of different ethnic background. Beside the main population of Hungarians the Roma (Gypsy/Romani) minority forms the largest ethnic group in Hungary with specific genetic background [15, 16]. Previous genetic studies revealed specific neuromuscular and other Mendelian disorders of the Roma people caused by private founder mutations [17, 18]. The unique and colorful population of Romani people is dispersed throughout the world. They are permanently migrating, however they live predominantly in Central and Eastern Europe. The major nation of Hungarians is genetically heterogeneous. This may reflect the ancient origin from the Eastern-Urals, important migratory routes throughout the history and the recent admixture with neighboring populations [19].

Accordingly, the genetic composition of Roma and Hungarian populations differ from the European Caucasians. Previous studies of several pharmacogenetically relevant genes already revealed differences in genetic structure of Roma and Hungarian populations [20–22]. Until now there is no report on the prevalence of the common *SLCO1B1* allelic variants in the constantly growing and world-wide spread populations of Roma people. The objective of this study was to determine the frequencies and haplotypes of *SLCO1B1* variant alleles in Roma and Hungarian populations, to compare our data to other populations, mainly to Indians and Caucasians studied. Population based screening can be used for risk stratification in prevention programs at a population level.

## Results and Discussion

The allele and genotype frequencies of the *SLCO1B1* A388G, T521C and T89595C polymorphisms in Roma and Hungarian populations are shown in Tables 1 and 2. The allele and genotype frequency distribution was in accordance with Hardy-Weinberg equilibrium in Roma and Hungarian subjects. The results of genotyping by PCR-RFLP were in concordance with direct sequencing of randomly selected wild type, heterozygous and homozygous variant samples.

**Table 1:** Genotype and allele frequencies of SLCO1B1 exonic polymorphisms in healthy Roma and Hungarian population samples compared to populations of different ethnic origin

Population	Number	G388A					T521C					Ref.
		Percent					Percent					
		AA^1^	AG	GG^1^	AG + GG^1^	G allele^1^	TT^2^	TC	CC	TC + CC	C allele	
Roma	470	24.5	42.1	33.4	75.5	54.5	67.0	31.5	1.49	33.0	17.2	Current study
Hungarian	442	45.5	36.6	17.9	54.5	36.2	65.2	31.9	2.94	34.8	18.9	Current study
Finnish	468	29.3	49.2	21.6	70.8	46.2	63.9	31.8	4.30	36.1	20.2	[26]
Indian (North)	270	31.9	46.7	21.4	68.1	45.0	-	-	-	-	-	[27]
Indian (Singapore)	100	17.0	52.0	31.0	83.0	57.0	87.0	13.0	0.00	13.0	6.50	[28]
Chinese (Singapore)	100	5.00	31.0	64.0	95.0	79.5	75.0	24.0	1.00	25.0	13.0	[28]
Chinese (Han)	111	9.00	35.1	55.9	91.0	73.4	73.8	24.3	1.80	26.1	14.0	[29]
Malays (Singapore)	100	2.00	22.0	76.0	98.0	87.0	79.0	20.0	1.00	21.0	11.0	[28]
Brazilian	143	55.9	35.7	8.40	44.1	26.2	74.1	23.8	2.10	25.9	14.0	[30]

Roma population is compared with Hungarians: ^1^ *p* < 0.001 and ^2^ *p* = 0.05

**Table 2:** Genotype and allele frequencies of SLCO1B1 intronic (T89595C) polymorphism in healthy Roma and Hungarian population samples compared to population data from the HapMap project

Population	Number	T89595C					Ref
		Percent					
		TT	TC	CC	TC + CC	C allele	
Roma	470	308 (65.5)	150 (31.9)	12 (2.60)	162 (34.5)	0.185	Current study
Hungarian	442	285 (64.5)	141 (31.9)	16 (3.60)	157 (35.5)	0.196	Current study
European (CEU)	113	77 (68.1)	35 (31.0)	1 (0.90)	36 (31.9)	0.164	HapMap project
Italian	102	60 (58.8)	38 (37.3)	4 (3.90)	42 (41.2)	0.225	
Indian (Gujarati from Houston)	101	88 (87.1)	13 (12.9)	0 (0.00)	13 (12.9)	0.064	
Japanese (Tokyo)	113	44 (38.9)	49 (43.4)	20 (17.7)	69 (61.1)	0.394	
Chinese (Han)	135	44 (32.6)	60 (44.4)	31 (23.0)	91 (67.4)	0.452	
Chinese (Colorado)	108	34 (31.5)	44 (40.7)	30 (27.8)	74 (68.5)	0.481	
African (USA)	57	36 (63.2)	18 (31.6)	3 (5.30)	21 (36.9)	0.211	
Kenya (Luhya)	109	78 (71.6)	29 (26.6)	2 (1.80)	31 (28.4)	0.151	
Kenya (Maasai)	156	106 (67.9)	43 (27.6)	7 (4.50)	50 (32.1)	0.183	
Nigeria (Yoruban)	147	109 (74.1)	36 (24.5)	2 (1.40)	38 (25.9)	0.136	
Mexican (LA)	57	46 (80.7)	10 (17.5)	1 (1.80)	11 (19.3)	0.105	

No statistical difference was explored when Roma population was compared to Hungarians

Significant differences were found comparing the Roma and Hungarian populations in the *SLCO1B1* 388 AA (24.5 vs. 45.5 %), GG (33.4 vs. 17.9 %) genotypes, AG + GG (75.5 vs. 54.5 %) carriers and in G allele frequency (0.545 vs. 0.362) (*p* < 0.001), respectively. Furthermore, the frequency of *SLCO1B1* 521 TT (67.0 vs. 65.2 %) was significantly higher in Roma than in Hungarian population samples (*p* = 0.05). No significant difference was observed in *SLCO1B1* T521C and the rs4363657 genotypes and minor allele frequencies between the Roma and Hungarian population samples.

The major haplotypes (ht) created by the examined SLCO1B1 variants are shown in Table 3. The *SLCO1B1* haplotype frequencies of Roma and Hungarians are summarized in Table 4. The most common *SLCO1B1* haplotype was the ht8 (GTT) both in Roma (43.6 %) and in Hungarian (59.1 %) samples. The ht6 (GCT) was not present in Roma population samples. Haplotype analyses showed striking differences between the Roma and Hungarian samples in ht4 (ATT, 37.2 % vs 20.8 %), ht5 (GCC, 1.15 % vs. 3.62 %) and ht8 (GTT, 43.6 % vs. 59.1 %) haplotypes (*p* < 0.01), respectively.

**Table 3:** Major haplotypes (ht) created by the examined SLCO1B1 variants

	rs2306283	rs4149056	r4363657
ht1	A	C	C
ht2	A	C	T
ht3	A	T	C
ht4	A	T	T
ht5	G	C	C
ht6	G	C	T
ht7	G	T	C
ht8	G	T	T

**Table 4:** Haplotype frequencies of the examined SLCO1B1variants in Roma and Hungarian population samples

	Roma (%)	Hungarian (%)
ht1	15.4	14.7
ht2	0.66	0.39
ht3	1.19	0.32
ht4^a^	37.2	20.8
ht5^a^	1.15	3.62
ht6	-	0.18
ht7	0.74	0.93
ht8^a^	43.6	59.1

^a^Roma population is compared with Hungarians: *p* < 0.01

**Fig. 1:**
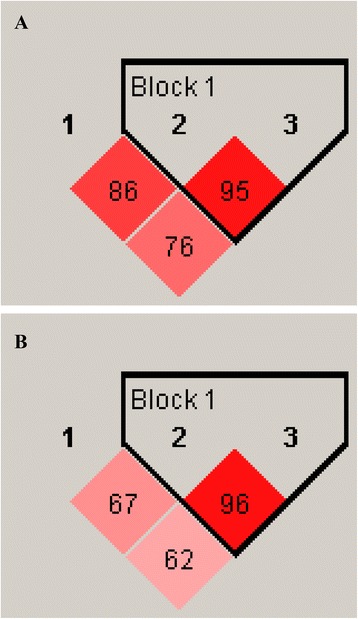
Linkage disequilibrium analysis for the SLCO1B1 rs2306283 (1), rs4149056 (2) and rs4363657 (3) polymorphisms in Roma (**a**) and Hungarian (**b**) populations

Linkage disequilibrium analysis of SLCO1B1 rs2306283, rs4149056 and rs4363657 variant alleles showed that the studied alleles are in different linkage disequilibrium patterns according to the different ethnic origin of Roma and Hungarian populations (Fig. 1). The rs4149056 and rs4363657 polymorphisms were in nearly complete linkage disequilibrium both in Roma (LD = 95) and Hungarian (LD = 96) populations. In addition, the Roma group presented also high degree of linkage disequilibrium in SLCO1B1 rs2306283and rs4149056 SNPs (LD = 86).

Statins are used as prophylactic treatment to reduce the risk of cardiovascular events. Statins have high inter-individual pharmacokinetic variability, up to 10 fold or more [23, 24].

The allele and genotype frequencies of *SLCO1B1* gene variants are dependent on ethnicity. Up to 2–5 % of individuals in various populations may be expected to show markedly elevated plasma pravastatin concentrations due to the *SLCO1B1* polymorphisms [1]. There are several disease conditions which have been found to be associated with these polymorphisms in *SLCO1B1* gene. The prevalence of *SLCO1B1* genotypes and allele frequencies reported from different ethnic populations residing in different geographic areas are summarized in Tables 1 and 2.

Similarly to other Caucasian populations the *SLCO1B1* 388G allele (rs2306283) is the minor allele in Hungarians, however in Roma is the 388A was found to be the minor allele, like in Indians from Singapore, however, not in indigenous Indians from North of Indian subcontinent (Table 1). The individuals that were homozygous for the G allele of rs2306283 and glucose 6-phosphate-dehydrogenase deficient were more frequent among the neonatal hyperbilirubinemia cases [25].

The allele and genotype frequencies of *SLCO1B1* rs4363657 noncoding polymorphism in Roma and Hungarian populations compared with data from the HapMap project are shown in Table 2. The observed 89595C allele frequency in Hungarians is slightly elevated compared to Roma and average European population (CEU) (http://hapmap.ncbi.nlm.nih.gov/). Interestingly, the 89595C allele frequency measured in Roma samples is almost three-fold higher than in Indians (Gujarati) and nearly similar to data was found in Africans. When comparing to other non-HapMap data, the C allele frequency of rs4363657 SNP in Hungarian (19.6 %) and Roma (18.5 %) populations found in our study was similar to that reported for Caucasian subjects, 15.4 % [26] and 13.0 % (GWAS study) [9]. Carrying the 89595C intronic variant means markedly elevated exposure to simvastatin and increased risk to myopathy [12, 27]. The odds ratio of SLCO1B1 rs4363657 for myopathy was 4.5 (95 % confidence interval 2.6-7.7) per copy of the C allele, and 16.9 (95 % confidence interval 4.7-61.1) in CC as compared with TT homozygotes. Genomic typing may allow the identification of these variants, leading to a tailored statin therapy with higher benefits to the patients and less adverse side effects [27].

Interestingly, regarding *SLCO1B1* rs2306283 SNP, Melo et al. found significant differences in allele frequencies when compared European Portugal population with the data of HapMap CEU population [28]. Furthermore, in a recent study investigated European Czech patients were treated with low statin doses there was no association found between rs4363657 SNP in *SLCO1B1* and risk of myalgia/myopathy [29].

The *SLCO1B1* T521C SNP (rs4149056) is common in non-African populations: Caucasian 8–20 %, Chinese 16 %, Japanese 10–16 % (Table 1). The minor allele frequency of T521C SNP is almost three times higher in Roma than in Indians (Singapore). Furthermore, the Roma population differs also from Hungarians and Caucasians in prevalence of this polymorphism. The results of *SLCO1B1* polymorphisms found in the Hungarian population were similar to that observed in other Caucasian populations. The 521C variant representing *SLCO1B1* haplotype (*15) is associated with rifampin-induced liver injury [30]. This non-synonymous coding SNP in *SLCO1B1* markedly increases systemic exposure to simvastatin and the risk of muscle toxicity. The strength of the evidence is high for myopathy with simvastatin [14].

The most common *SLCO1B1* haplotype was ht8 both in Roma and Hungarian populations. In both examined populations apart from ht8, the most frequent haplotype was ht4 (containing neither variant) followed by ht1 (including 521C and 89595C intronic variants). The ht6 was present in Hungarians with a low frequency (0.18)**,** but was not detectable in Roma samples. Ht2, representing the T521C variant, is associated with decreased transporter activity that may potentially be associated with increased statin efficacy.

## Conclusions

If we consider, these results suggest that the pharmacogenetical pattern of *SLCO1B1* gene shows population specificity; therefore the examination of clinically relevant polymorphisms is important in different populations. The phenotype effect of the *SLCO1B1* haplotypes on the transporter activity need to be investigated in future clinical studies of these populations, as well as the effect on statin treatment. Comparing the LD patterns of the two studied populations we can conclude, Roma samples showed higher degree of linkage disequilibrium of studied SNPs than Hungarians. As both of the studied populations present high degree of LD patterns, haplotypes analyses of *SLCO1B1* gene exerts less advantage, especially because only the rs4149056 polymorphism have been described as clinically functional.

## Methods

### Study population

The DNA samples were from the central Biobank of the University of Pecs that is part of the National Biobank Network of Hungary, as well as the pan-European Biobanking and Biomolecular Resources Research Infrastructure (BBMRI) (http://bbmri-eric.eu/). The governance principles and maintenance management of the Biobank had been approved by the Hungarian National Research Ethics Committee (ETT TUKEB, Budapest, Hungary). During the collection and analysis of DNA samples and processing of the accompanying personal data the guidelines and regulations of the Helsinki Declaration in 1975 and the currently operative National regulations were followed. DNA of total of 470 healthy Roma samples (170 males and 300 females, mean age 39 ± 16 years, range: 18–93 years) and 442 healthy Hungarian (183 males and 259 females, mean age 45 ± 10 years, range: 18–66 years) were used in the study. Informed consent was obtained from all subjects.

### Molecular methods

Genomic DNA was isolated from peripheral leukocytes using routine salting out method, and was store with the patient’s informed consent. Three polymorphisms of the *SLCO1B1* (MIM*604843, 12p12) gene including the rs2306283 (A388G, N130D, exon 5), the rs4149056 (T521C, V174A, exon 5) and rs4363657 (T89595C, intron 11) were analyzed. For primer design the sequences deposited into the GenBank (http://www.ncbi.nlm.nih.gov/genbank/) were used.

Genotyping was carried out using polymerase chain reaction (PCR) followed by restriction endonuclease digestion (RFLP). For detection of the rs2306283 the following primers were used: 5’-CTGTGTTGTTAATGGGCGAA-3’ and 5’-GGGGAAGATAATGGTGCAAA-3’. The rs4149056 SNP was detected using the following set of primers: 5'- TTGTCAAAGTTTGCAAAGTG -3’ forward and 5’- GAAGCATATTACCCATGAGC -3’ reverse. And the rs4363657 was detected with the help of the following primers: 5’-CAGTTTGCTAGTGTTTTGTTGAGG-3’ forward, 5’-ACCATCCAAGACGAACAAAGAG -3’ reverse. Underlining in the sequence indicates mismatch base introduced to generate artificial cleavage site. PCR amplification was carried out in a final volume of 50 μl on an MJ Research PTC 200 thermal cycler. PCR conditions were as follows: predenaturation for 2 min at 96 °C, followed by 30 cycles of denaturation for 30 s at 95 °C, annealing for 1 min by primer specific temperature, primer extension for 30 s at 72 °C, the final extension at 72 °C for 5 min. Annealing temperature for the A388G, T521C and T89595C primers were 57, 52 and 55 °C, respectively.

Ten μl PCR products of the A388G, T521C and T89595C primers was digested by Taq1, Hin6I and KpnI restriction enzymes, respectively. Supplier of the restriction endonucleases is the Thermo Scientific, the incubation temperature was 65, 37 and 37 °C, respectively. The digested PCR products were separated by electrophoresis using a 3 % agarose gel stained with ethidium bromide and visualized by an UV transilluminator. In the amplicons of the A388G polymorphism was an obligatory cleavage site to enable us to monitor the efficacy of the digestion. In the samples with 388 AA genotype the Taq1 cleaves the 406-bp long PCR product in 159-bp and 247-bp fragments. If the 388 G allele was present in homozygouse form the 23-, 136-, 247-bp fragments could be detected. The 209-bp amplicon of the T521C SNP in the samples with CC genotype was digested into 21-bp and 188-bp long fragments. While in TT homozygotes the recognition site of the enzyme was missing, resulting in no digestion. However, all the homozygote variant samples from the PCR-RFLP assay were direct sequenced with the same primers utilizing an ABI 3500 genetic analyzer. Wild-type of 89595 TT yielded two fragments of 133 bp and 236 bp in length, and the variant 89595 CC homozygotes gave three fragments of 133 bp, 84 bp and 152 bp. The heterozygotes formed four fragments of 84 bp, 133 bp, 152 bp and 236 bp. The accuracy of the genotyping was evaluated by direct sequence analysis of randomly selected samples as described above.

### Statistical analysis of data

The method used for calculation of the Hardy-Weinberg equilibrium in different SNPs was described by G. H. Hardy and W. Weinberg in 1908 [31].

Statistical significance (*p* < 0.05) was assessed by χ^2^ test to compare the differences between studied groups. Statistical analyses were performed applying Excel for Windows and SPSS 11.5 package for Windows (SPSS Inc., Chicago, IL).

Haploview 4.1 was used to study linkage disequilibrium (LD) patterns. We required the minor allele frequency at each locus to be >0.05, with an R^2^ value of <0.8 between pairs of loci, based on the default settings in Haploview. Haplotype frequencies were estimated using PHASE version 2.1 [32, 33]. The allele composition of major haplotypes (ht) created by the examined *SLCO1B1* variants is shown in Table 3.

### Availability of supporting data

The identification numbers of the examined SNPs in *SLCO1B1* gene (Uniprot identifier: Q9Y6L6 is available at http://www.uniprot.org/uniprot/Q9Y6L6) for c.521T > C, c.388A > G and c.1498-1331T > C polymorphisms were rs4149056, rs2306283 and rs4363657, respectively based on the dbSNP database of NCBI (National Center for Biotechnology Information-http://www.ncbi.nlm.nih.gov/SNP/).

## Abbreviations

SLCO1B1: Solute carrier organic anion transporter family member 1B1
OATP1B1: Organic anion transporting polypeptide 1B1
PCR: Polymerase chain reaction
RFLP: Restriction fragment length polymorphism
SNP: Single nucleotide polymorphism
ht: Haplotype
LD: Linkage disequilibrium
CVD: Cardiovascular disease
FDA: Food and drug Administration
CPIC: Clinical Pharmacogenetics Implementation Consortium
BBMRI: Biobanking and Biomolecular Resources Research Infrastructure
